# Higher Neutrophil-to-Lymphocyte Ratio and Systemic Immune–Inflammation Index Is Associated with Better Prognosis Following Pancreaticoduodenectomy for Pancreatic Adenocarcinoma

**DOI:** 10.3390/jcm14113762

**Published:** 2025-05-27

**Authors:** Esther Kazlow, Elias Rinawi, Eden Gerszman, Samar Mattar, Nabih Essami, Mary Nasir, Aasem Abu Shtaya, Wisam Assaf, Riad Haddad, Ahmad Mahamid

**Affiliations:** 1 Technion Israel Institute of Technology, Rappaport Faculty of Medicine, Haifa 3109601, Israel; ekazlow@gmail.com (E.K.); rinawi.elias@gmail.com (E.R.); eden.gerszman@gmail.com (E.G.); samar.mattar11@gmail.com (S.M.); nabih4e@gmail.com (N.E.); maryna@clalit.org.il (M.N.); asemab@clalit.org.il (A.A.S.); wissam_assaf_90@hotmail.com (W.A.); dr.riad.haddad@gmail.com (R.H.); 2 Department of Surgery, Carmel Medical Center, Michal Str. 7, Haifa 3436212, Israel; 3 Department of Gastroenterology, Carmel Medical Center, Haifa 3436212, Israel; 4 Department of Obstetrics and Gynecology, Carmel Medical Center, Haifa 3436212, Israel

**Keywords:** pancreatic cancer, neutrophil-to-lymphocyte ratio (NLR), systemic immune–inflammation index (SII), prognostic nutritional index (PNI), pancreaticoduodenectomy, survival outcomes, complications

## Abstract

**Background:** Pancreatic cancer has a high mortality rate worldwide. Most patients progress to advanced stages, often with metastasis, resulting in a low survival rate. Despite advancements in surgical and oncological treatments, early diagnosis and better risk stratification remain critical. **Methods**: This retrospective cross-sectional study focused on analyzing data from patients undergoing pancreaticoduodenectomy for pancreatic adenocarcinoma, in order to determine whether the neutrophil-to-lymphocyte ratio (NLR) and other immune–inflammatory markers, such as the systemic immune–inflammation index (SII) and prognostic nutritional index (PNI), can predict postoperative complications and survival outcomes. **Results**: Analysis of 136 patients revealed that a higher NLR (≥2.5) was significantly associated with longer overall survival (39 months, IQR: 17–100 months; *p* = 0.004), compared to lower NLR (<2.5; 18 months, IQR: 9–39 months). Higher SII (≥600) was also associated with significantly improved survival (34 months, IQR: 17–114 months; *p* = 0.001) compared to lower SII (<600; 20 months, IQR: 9–45 months). No significant differences were observed in postoperative complications or other clinical outcomes between NLR groups, although a trend toward more complications in the higher NLR group was noted (*p* = 0.06). PNI showed no significant impact on survival (PNI < 38.8: 22 months, IQR: 14–60 months; PNI ≥ 38.8: 33 months, IQR: 14–115 months; *p* = 0.1) or complications (*p* = 0.8). **Conclusions**: Our study highlights the prognostic utility of NLR and SII in patients with adenocarcinoma of the head of the pancreas undergoing pancreaticoduodenectomy. Regarding complications, there were no significant differences across groups stratified by NLR, SII, or PNI, suggesting that while NLR and SII are valuable for predicting long-term oncological outcomes in patients undergoing pancreaticoduodenectomy for adenocarcinoma of the head of the pancreas, they may not be reliable indicators of immediate postoperative morbidity.

## 1. Introduction

Pancreatic carcinoma has one of the highest mortality rates among all solid organ cancers [1]. In 2019, it was estimated that 56,770 people were diagnosed with pancreatic cancer, and 45,750 deaths occurred as a result of this in the United States [2]. The incidence of pancreatic cancer varies significantly between countries. The highest age-standardized incidence occurred in Europe and North America, while the lowest age-standardized incidence occurred in Africa and South-Central Asia [3]. Pancreatic cancer-related deaths are projected to increase, making it the second leading cause of cancer-related deaths in the United States by 2030, up from its current position as the fourth [4,5]. According to a 2018 study that analyzed data from the United States Surveillance, Epidemiology, and End Results Program (SEER), between 1973 and 2014, the age-standardized incidence rates of pancreatic cancer have increased by 1.03% annually [6].

The majority of pancreatic cancers are diagnosed at a late stage, either as locally advanced tumors or with metastatic disease, due to the lack of early diagnostic testing modalities. The anatomical location of the pancreas makes surgery challenging due its close proximity to the main vascular structures, and about 80% of tumors are not resectable at the time of presentation [7]. In cases where surgery is possible, surgical resection is the only treatment that provides a potential cure [8].

Pancreaticoduodenectomy and distal or total pancreatectomy, are the primary surgical options for resecting pancreatic cancer, with the choice depending on the anatomical location of the tumor. While adjuvant treatment plans can improve survival, 71% to 76% of patients experience relapse within two years, and nearly 40% are unable to proceed to adjuvant therapy due to surgical complications [9]. Given the high rates of relapse and complications, there is an urgent need for better preoperative prognostic markers to predict surgical outcomes and better inform treatment decisions for patients undergoing pancreatic cancer surgery.

The TNM classification system is commonly used as a reliable prognostic tool for cancer patients. However, since it incorporates pathological tumor findings, a definitive classification can only be determined after surgery. Thus, there is increasing interest in preoperative prognostic indicators, particularly blood-based biomarkers, as they offer the potential for more accurate patient stratification. These biomarkers can help guide clinical decision-making and improve both short- and long-term outcomes postoperatively [10]. Precisely how different biomarker levels might alter specific treatment strategies in the unique context of pancreaticoduodenectomy warrants further investigation.

Blood-based biomarkers offer a convenient and objective approach to clinical risk stratification. The neutrophil-to-lymphocyte ratio (NLR) is a biomarker linked to systemic inflammation and poor oncologic prognosis. Elevated NLR has been identified as a potential predictor for adverse surgical outcomes, as associated immunologic and coagulation disturbances can lead to many postoperative complications such as sepsis. Research has demonstrated that the NLR helps identify patients at higher risk for perioperative complications following surgery for solid tumors, including esophageal, lung, gastric, bladder, and colorectal cancers [9,10,11,12,13,14]. Furthermore, the systemic immune–inflammation index (SII), another inflammatory biomarker [calculated as (absolute platelet count × absolute neutrophil count)/lymphocyte count], has shown to have significant prognostic value in several types of cancer, including pancreatic cancer, renal cell carcinoma, and gastro-esophageal cancer [15,16,17,18]. The prognostic nutritional index (PNI) is yet another blood-based biomarker that is easily calculated using the formula [(10 × serum albumin (g/dL)) + (0.005 × total lymphocyte count)]. The components of this index—serum albumin and lymphocyte count—are commonly assessed in routine preoperative lab tests and are simple to monitor over time. The PNI’s predictive value for surgical outcomes is well-established across various solid organ cancers, including those of the esophagus, colon, and liver [19,20,21,22].

Although NLR, SII, and PNI have been investigated in patients diagnosed with pancreatic cancer, their association with outcomes in patients specifically undergoing pancreaticoduodenectomy for pancreatic adenocarcinoma remains largely unexplored. Most of the research to date has focused on the general pancreatic cancer population, with very few publications exploring the role of these inflammatory markers in patients undergoing surgical intervention, specifically pancreaticoduodenectomy [23,24].

Wang et al. [25] conducted a systematic review and meta-analysis which found that elevated preoperative NLR is associated with diminished overall survival (OS) and disease-free survival (DFS) in patients undergoing pancreaticoduodenectomy (PD). Elevated NLR was also identified as an independent predictor of immediate postoperative complications. However, these analyses included heterogeneous populations with varying tumor locations, histology, surgical procedures, and treatment sequences. There is a paucity of data specifically addressing patients with adenocarcinoma of the pancreatic head undergoing upfront pancreaticoduodenectomy, a group with distinct clinical characteristics and management considerations. Our study aims to address this gap by focusing exclusively on this well-defined cohort.

The unique challenges faced by patients undergoing pancreaticoduodenectomy, including postoperative recovery, surgical trauma, and the potential for complications—may significantly influence the inflammatory environment in ways that are not reflected in studies that consider a broader group of pancreatic cancer patients. This specific surgical group could experience a distinct inflammatory response due to factors such as tissue injury, infection, or the physiological stress associated with the procedure itself. Additionally, altered immune function post-surgery might impact the inflammatory markers, which could lead to different prognostic outcomes compared to those observed in nonsurgical cohorts. Therefore, the lack of extensive research on this subset may account for the variability in findings from existing studies, highlighting the need for more targeted investigation into how these inflammatory markers behave and predict outcomes in patients following pancreaticoduodenectomy for pancreatic adenocarcinoma.

By better understanding these associations, the aim is to refine postoperative care, improve prognostic assessment, and potentially define clearer links to specific clinical pathways for this patient population. The establishment of reliable preoperative prognostic biomarkers has significant implications for clinical decision-making in pancreatic adenocarcinoma management. These biomarkers could guide several critical decisions, including surgical approach selection, perioperative care planning, prioritization for adjuvant therapy, patient counseling, and clinical trial stratification. Therefore, identifying accessible and reliable biomarkers such as NLR, SII, and PNI could significantly impact treatment planning and resource allocation in this challenging patient population.

Building on these findings, our study analyzed data from patients undergoing pancreaticoduodenectomy for adenocarcinoma of the head of the pancreas to assess whether the NLR, SII, and PNI can predict postoperative complications and survival outcomes.

## 2. Materials and Methods

Patients who underwent pancreatectomy between January 2008 and June 2023 were identified from the surgical databases at Carmel Medical Center located in Haifa, Israel. This university-affiliated tertiary care hospital is renowned for its extensive hepato-biliary unit and over two decades of experience in advanced hepato-pancreato-biliary surgery. The data were collected from the Chameleon database, a comprehensive electronic medical record system, which includes detailed patient information and perioperative data. The study was conducted in accordance with the Declaration of Helsinki and Good Clinical Practice Guidelines and was approved by the institutional review board (IRB) of Carmel Medical Center. Our review included consideration of patient demographics, detailed surgical history, and follow-up from the medical/oncology team. All patients received anesthesiologic clearance and approval from a multidisciplinary surgical team. All patients were informed about the procedure, including risks and benefits. Written consent for surgery was obtained from all patients. Due to the retrospective nature of this study, informed consent from patients was waived by the Institutional Review Board Committee.

### 2.1. Study Population

The study population consisted of patients aged 18–90 years with resectable pancreatic head adenocarcinoma who underwent upfront open pancreaticoduodenectomy followed by adjuvant therapy [26]. Patients were excluded if they had non-adenocarcinoma histology, underwent non-curative (palliative) surgery or procedures other than pancreaticoduodenectomy, had metastatic disease at the time of surgery, had incomplete clinical or laboratory data necessary for calculation of NLR, SII, or PNI, were younger than 18 or older than 90 years, or had pre-existing active infections, autoimmune diseases, or other inflammatory conditions that could confound immune–inflammatory marker levels. During the study period, 310 patients underwent pancreatectomy, of whom 136 met these criteria and were included in the analysis.

### 2.2. Outcomes

The association between blood-based biomarkers with postoperative complications and overall survival was assessed using logistic regression.

Our study’s cutoff values for NLR (2.5), SII (600), and PNI (38.8) were selected based on thresholds established in prior studies investigating their prognostic value in cancer [26,27,28]. These values are supported by research demonstrating their association with survival outcomes following surgical resection. We selected these cutoffs to ensure comparability with existing literature, while acknowledging that variations in these values across studies may contribute to differences in reported prognostic significance.

We analyzed the distribution of study variables to better understand the characteristics of the patient population using simple cross-tabulations for categorical variables and calculating their frequency distributions. The groups were compared in terms of perioperative outcomes and overall survival. Variables that could influence outcomes were assessed, including demographics as well as routine preoperative laboratory tests that included white blood cell, lymphocyte, platelet, neutrophil, hemoglobin, albumin, tumor markers (CA19-9, etc.), operative and postoperative parameters. Blood loss was estimated using the volume of blood aspirated from the abdominal cavity during the procedure. Operative time was defined as the time elapsed from the skin incision until closure. Length of stay was defined as the number of hospitalized days from the day of operation until the day of discharge, inclusive. Complications were defined as any unexpected event that deviated from a normal recovery course, occurring within 90 days of surgery. Severity of complications was graded using the Clavien–Dindo scoring system [29]. Postoperative pancreatic fistula (POPF) was defined according to the International Study Group on Pancreatic Fistulae (ISGPF) classification system, categorizing POPFs into biochemical (Grade A) and clinically significant (Grades B and C) fistulae. Grade A POPFs are defined as possessing measurable fluid output with elevated amylase levels on postoperative day 3, lacking significant clinical consequences. Clinically significant POPFs (Grades B and C) are characterized as follows. Grade B necessitates at least one of the following: endoscopic or radiological intervention, drain retention exceeding three weeks, clinical-symptom-absent organ failure, or a clinically relevant alteration in management. Progression to Grade C occurs with the implementation of major management changes, deviation from standard clinical pathways, or the manifestation of organ failure [30]. After discharge, all the patients were followed by our multidisciplinary team.

### 2.3. Statistical Analysis

The continuous variables were presented as mean ± standard deviation or as median with interquartile range (IQR), depending on their distribution. Categorical variables were expressed as percentages. A comparison of demographic and clinical characteristics between the patients in both groups was done using the chi-square test for the categorical variables and independent *t*-test or Mann–Whitney, as appropriate, for the continued variables. *p*-value of <0.05 was considered statistically significant. All statistical analyses were conducted using International Business Machines Corporation (IBM, New York, NY, USA) SPSS Statistics, version 24.

In this study, we did not perform a multivariate analysis including all three indices (NLR, SII, and PNI) simultaneously. Our primary analytic approach focused on evaluating the prognostic value of each index individually using univariate analyses. This decision was based on both methodological considerations and the sample size of our cohort. We acknowledge that future studies with larger populations may be able to further explore the independent and combined prognostic value of these indices using multivariate modeling.

## 3. Results

Table 1 presents the characteristics of 136 patients, divided into two groups based on their neutrophil-to-lymphocyte ratio (NLR): those with NLR < 2.5 (36%) and those with NLR ≥ 2.5 (64%). Results show that there was no significant difference between the two groups in terms of age (*p* = 0.46), ethnicity (*p* = 0.36), hypertension (*p* = 0.87), diabetes mellitus (*p* = 0.64), ischemic heart disease (*p* = 0.75), smoking status (*p* = 0.14), levels of CA19.9 (*p* = 0.92), and PNI (*p* = 0.68). However, a significant difference was found between the groups in terms of SII (*p* = 0.001) and sex (*p* = 0.03).

**Figure 1:**
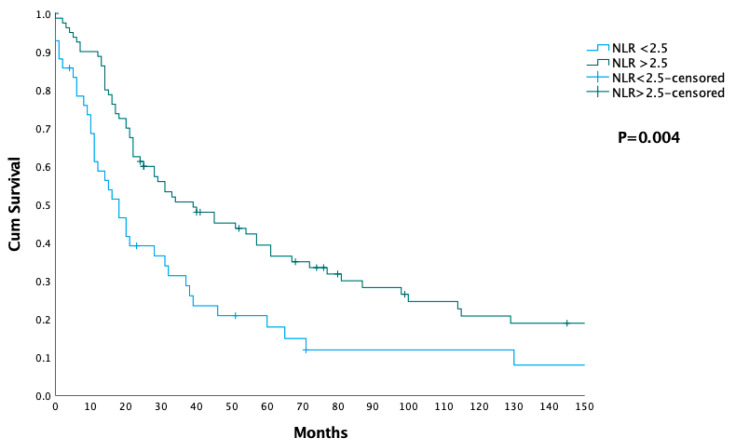
Survival analysis in patients stratified by their neutrophil-to-lymphocyte ratio (NLR), with two groups: patients with NLR < 2.5 (blue line) and NLR ≥ 2.5.

Table 2 presents the operative and postoperative outcomes for patients categorized by NLR into two groups: NLR < 2.5 and NLR ≥ 2.5. There was no significant difference in the average operating room time (*p* = 0.89), intraoperative blood loss (*p* = 0.6), the percentage of patients requiring ICU admission (*p* = 0.155), and the average duration of ICU stay (*p* = 0.43). Although patients with NLR ≥ 2.5 showed a trend toward more frequent postoperative complications (59.8%) compared to the NLR < 2.5 group (40.2%), this difference did not reach statistical significance (*p* = 0.06). The severity of postoperative complications, classified by the Clavien–Dindo scale, did not show a significant difference between the groups (*p* = 0.22). There was no significant difference in the incidence or severity of postoperative pancreatic fistulas between the groups (*p* = 0.6). Patients in the NLR < 2.5 group had a slightly longer average hospital stay (21.3 ± 15.8 days) compared to those in the NLR ≥ 2.5 group (17.4 ± 12.2 days), but this difference was not statistically significant (*p* = 0.123). The Kaplan–Meier survival curve compares the cumulative survival between the two groups of patients based on their NLR (Figure 1). Results show that the median overall survival for patients with NLR < 2.5 was 18 months (IQR: 9–39 months), while patients with NLR ≥ 2.5 had a significantly longer median overall survival of 39 months (IQR: 17–100 months). This difference in survival between the groups is statistically significant, with a *p*-value of 0.004 (Table 2) (Figure 1).

**Figure 2:**
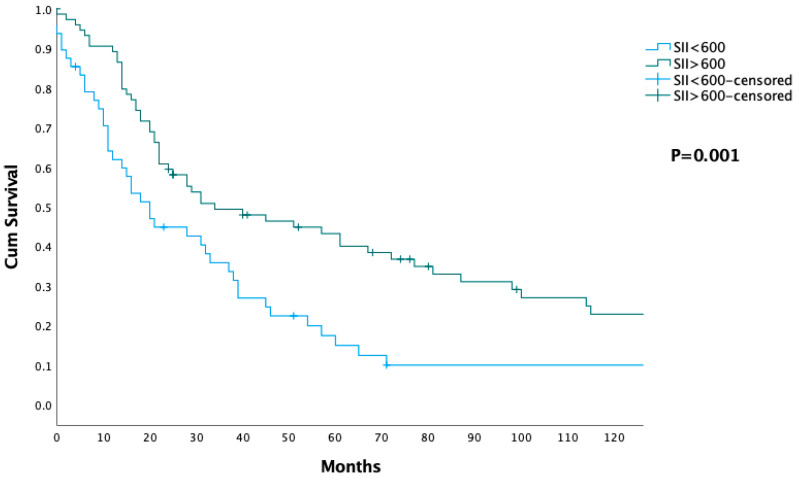
Survival analysis in patients stratified by their wystemic immune–inflammation index (SII), with two groups: SII < 600 (blue line) and SII ≥ 600.

The Kaplan–Meier survival curve illustrates cumulative survival in patients stratified by their SII, with two groups: SII < 600 and SII ≥ 600 (Figure 2). It reveals that patients with SII < 600 experience significantly worse survival outcomes compared to those with SII ≥ 600. Patients in the SII ≥ 600 group exhibit better long-term survival, with a substantial proportion of patients surviving beyond 100 months, while the survival rates in the SII < 600 group sharply decline within the first 50 months. The median overall survival (OS) for the SII < 600 group is 20 months (IQR: 9–45 months), while the SII ≥ 600 group has a significantly longer median OS of 34 months (IQR: 17–114 months) (*p* = 0.001) (Table 3).

In terms of postoperative complications, the data show that patients with SII < 600 experienced a lower complication rate (44%) compared to those with SII ≥ 600 (56%). Although there is a trend suggesting higher complication rates in the SII ≥ 600 group, this difference is not statistically significant (*p* = 0.07). The severity of complications, based on the Clavien–Dindo classification, does not show a statistically significant difference between the groups (*p* = 0.1) (Table 3).

**Figure 3:**
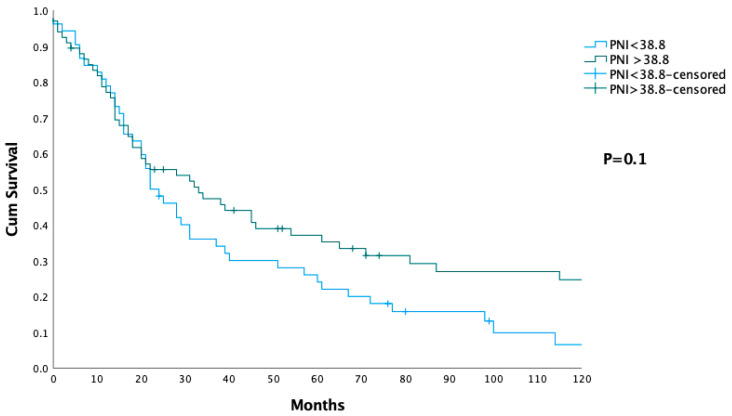
Survival analysis in patients stratified by their prognostic nutritional index (PNI), with two groups: patients with PNI < 38.8 (blue line) and PNI ≥ 38.8.

The Kaplan–Meier survival curve presents cumulative survival based on the PNI in two groups: patients with PNI < 38.8 and PNI ≥ 38.8 (Figure 3). Results show that the median OS for patients with PNI < 38.8 was 22 months (IQR: 14–60 months), while the median OS for patients with PNI ≥ 38.8 was 33 months (IQR: 14–115 months). However, this difference in survival did not reach statistical significance (*p* = 0.1). The complication rates were comparable between the two groups, with 45% of patients in the PNI < 38.8 group and 55% of patients in the PNI ≥ 38.8 group experiencing complications. There was no significant difference in complication rates between the groups (*p* = 0.8). Moreover, the severity of complications, according to the Clavien–Dindo classification, did not differ significantly between the groups (*p* = 0.5) (Figure 3) (Table 4).

## 4. Discussion

This study found a statistically significant association between higher NLR and improved overall survival, with patients in this group exhibiting a significantly longer median survival time. Similarly, a higher SII was associated with better overall survival. In contrast, PNI did not show a statistically significant association with overall survival. Regarding postoperative complications, while a trend towards higher complication rates was observed in the higher NLR group, this difference did not reach statistical significance. Similarly, no significant difference in complication rates or severity was observed between SII or PNI groups.

Pancreaticoduodenectomy and distal or total pancreatectomy are the primary surgical options for resecting pancreatic cancer, depending on the tumor’s anatomical location. Achieving an R0 resection is crucial, as it significantly improves survival compared to R1 resections. To increase the rate of microscopic clearance, neoadjuvant treatments and vascular resections have been employed [31]. Adjuvant therapies have been found to be beneficial in terms of improving overall survival; however, a significant percentage of patients experience relapse within a couple of years, and many are unable to proceed with treatment due to surgical complications. This has prompted interest in neoadjuvant therapies, which may help reduce recurrence by shrinking the tumor and eliminating micrometastases [32,33]. To further improve outcomes, there is a growing need for preoperative prognostic markers, especially blood-based biomarkers, that can guide clinical decisions. Biomarkers such as the NLR, SII, and PNI have shown potential in predicting surgical complications and survival, offering a more objective and accessible way to assess patient risk before surgery.

Previous studies have demonstrated the utility of NLR as a prognostic tool in various cancers. Yuchen et al. found that an NLR <4 was associated with improved disease control in patients with colorectal cancer and synchronous liver metastasis (*p* = 0.024). Additionally, elevated NLR was identified as a significant predictor of poor OS and progression-free survival (PFS) (*p* = 0.027) [34]. A study by Mao et al. further highlighted the predictive value of NLR in patients undergoing preoperative chemotherapy for colorectal liver metastases, showing that a pre-treatment NLR of 2.4 ± 1.1 was significantly associated with pathological response following resection. Specifically, patients with an NLR < 2.3 had a pathological response rate of 67%, compared to a 48.1% response rate in those with an NLR > 2.3 (*p* = 0.01) [35]. A study by Cupp et al. analyzed 204 meta-analyses from 86 studies to assess the association between NLR and cancer outcomes. Most studies found that elevated NLR was linked to worse cancer prognosis, with 18 associations showing strong evidence across various cancer types, particularly urinary, nasopharyngeal, and gastric cancers [36]. Contrary to previous studies suggesting that elevated NLR signals poor prognosis due to its association with chronic inflammation, a study by Li et al. found that high NLR might be protective and associated with better prognosis in hepatocellular carcinoma. The authors suggest that neutrophils, which are elevated in high NLR levels, may help eliminate mutated cells and promote anti-tumor immunity through increased activation of T-cells. They also noted that neutrophils are key players in the tumor microenvironment, supporting immune responses and aiding tumor defense [37].

SII has also emerged as a valuable prognostic factor in certain cancers, helping to predict patient outcomes postoperatively. A meta-analysis by Xiaoqu Li et al. evaluated the prognostic value of SII in esophageal squamous cell carcinoma (ESCC) patients undergoing surgery. After analyzing nine retrospective studies involving 3565 patients, the results showed that a higher SII was significantly associated with poorer OS, PFS, and cancer-specific survival (CSS) in ESCC patients. Specifically, high SII was an independent predictor of worse OS in ESCC patients (HR = 1.72) [17]. A study by Chen et al. investigated the clinical significance of the preoperative SII in predicting outcomes for colorectal cancer (CRC) patients undergoing radical surgery. The researchers found that higher SII was associated with worse overall survival and disease-free survival (DFS). Compared to other inflammation-based markers such as the NLR and platelet–lymphocyte ratio (PLR), SII showed superior predictive value, with a higher area under the curve for both OS and DFS. SII was identified as an independent prognostic factor and outperformed NLR and PLR in discriminating survival outcomes across different TNM stages [38].

One of the main findings of our study was the significant impact of both the NLR and SII on survival. The Kaplan–Meier survival curve demonstrated a marked difference in overall survival between patients with NLR < 2.5 and those with NLR ≥ 2.5, with patients having higher NLR values showing significantly better survival outcomes—39 months compared to 18 months in the lower NLR group (*p* = 0.004). Similarly, SII was another significant predictor of survival, with patients having SII ≥ 600 showing a median overall survival of 34 months, compared to 20 months for those with SII < 600 (*p* = 0.001). SII, which reflects a balance between inflammatory and immune responses by incorporating neutrophil, platelet, and lymphocyte counts, highlights a more favorable immune response that may help control tumor growth and dissemination [39]. While NLR and SII reflect different components of systemic inflammation, both markers typically trend in the same direction, as seen in our study, suggesting that they may reflect similar inflammatory responses within the tumor microenvironment [40]. Notably, while both markers were significant predictors of survival, neither NLR nor SII showed a statistically significant association with complication rates or severity (*p* = 0.06 and *p* = 0.07, respectively). These findings underscore that these immune–inflammatory markers are more indicative of long-term oncological outcomes than of immediate postoperative morbidity. Together, these results suggest that NLR and SII are valuable prognostic markers in patients undergoing pancreaticoduodenectomy for adenocarcinoma of the head of the pancreas, reflecting systemic tumor biology and immune status rather than perioperative complications.

While most existing literature including meta-analyses [25,41,42] have reported a consistent association between higher NLR and adverse prognosis in pancreatic cancer, our findings in a homogeneous cohort of patients undergoing upfront pancreaticoduodenectomy for pancreatic head adenocarcinoma suggest a different relationship. This discrepancy may be attributable to differences in patient selection, tumor biology, or perioperative factors unique to this group. Our results underscore the need for further research to clarify the prognostic utility of these indices in specific clinical contexts. We theorized that an elevated NLR and SII may reflect a stronger inflammatory response and enhanced immune surveillance, which could contribute to better tumor control and improved overall survival. A high NLR reflects an imbalance in the immune system, where neutrophils are more prevalent relative to lymphocytes. Similarly, a high SII indicates an altered immune–inflammatory status, with elevated neutrophils and platelets outnumbering lymphocytes. An increased neutrophil count can indicate a heightened inflammatory response, often associated with tissue injury, infection, or cancerous processes [43]. In the context of cancer specifically, a high NLR and SII may suggest that the body is mounting a strong inflammatory reaction in response to the tumor, but it can also signal a complex interplay between immune activation and immune evasion mechanisms employed by the tumor. In many cancers, the inflammatory environment within the tumor microenvironment plays a dual role. On the one hand, inflammation can promote tumor progression by supporting angiogenesis enhancing metastasis, and facilitating immune evasion by the tumor. On the other hand, a robust inflammatory response, indicated by a high NLR and/or a high SII, may also enhance immune surveillance. Neutrophils, although traditionally part of the innate immune system, can release pro-inflammatory cytokines and reactive oxygen species (ROS) that can indirectly influence adaptive immune cells such as T-lymphocytes. These activated lymphocytes may contribute to a stronger immune surveillance of the tumor, potentially increasing the effectiveness of the body’s immune response against cancer cells [44,45].

Our findings suggest that a higher NLR and SII are associated with improved survival in patients with adenocarcinoma of the head of the pancreas undergoing surgery. This association may be due to the fact that while neutrophils can promote inflammation, their activation may also enhance the anti-tumor effects of cytotoxic T-cells and natural killer (NK) cells, which are important components of adaptive immunity. The NLR and SII, thus, may serve as surrogate markers not only for inflammation but also for immune system engagement with the tumor [46]. Furthermore, the dynamics of NLR and SII can be indicative of the body’s ability to maintain a balance between inflammation and immune regulation. In cancers where a high NLR and SII correspond with better outcomes, this may reflect a situation where the inflammatory response is strong enough to facilitate immune-mediated tumor control, but not so excessive as to lead to immune suppression or the creation of a microenvironment that supports tumor growth. Essentially, the heightened immune activity represented by a high NLR and SII could be indicative of a “window” of effective immune surveillance, where the body is actively working to control the tumor without the immune system being overwhelmed or exhausted [47]. However, it’s also important to note that while a high NLR and SII can indicate improved immune response in certain cancers, they could also reflect a more aggressive disease state, where the immune system is struggling to overcome tumor-induced inflammation and immune evasion [48]. Therefore, NLR and SII should be interpreted in conjunction with other clinical and pathological markers to better understand their implications in cancer prognosis.

The apparent contradiction between our findings and those of previous studies may be explained by differences in study populations, clinical management, and methodological approaches. Our cohort consisted exclusively of patients with adenocarcinoma of the pancreatic head who underwent upfront pancreaticoduodenectomy, whereas many prior studies and meta-analyses included patients with tumors at various pancreatic sites, different histological subtypes, and those who received neoadjuvant therapy. These distinctions can influence both the tumor microenvironment and the systemic inflammatory response, potentially altering the prognostic impact of indices such as NLR, PNI, and SII. Furthermore, variability in the cut-off values used for these biomarkers, perioperative management protocols, and definitions of clinical endpoints may contribute to divergent findings. While we hypothesize that certain molecular mechanisms underlie the relationship between inflammatory indices and outcomes, it is likely that these mechanisms interact with clinical and methodological factors unique to each study population. Therefore, our results highlight the importance of context-specific research and suggest that the prognostic utility of these biomarkers may not be universally applicable across all patient groups and treatment settings.

The prognostic value of preoperative NLR and SII demonstrated in our study has several practical implications for clinical management. Patients with higher NLR and SII, who exhibit improved survival, may be considered for more aggressive surgical approaches and prioritized for adjuvant therapy. Conversely, patients with less favorable biomarker profiles may benefit from tailored perioperative care and closer postoperative surveillance. These biomarkers also provide clinicians with objective data to support patient counseling and shared decision-making. Additionally, NLR and SII may serve as useful criteria for stratifying patients in clinical trials, potentially identifying subgroups most likely to benefit from novel treatment modalities. Incorporating these biomarkers into routine preoperative evaluation could therefore enhance individualized care and optimize both short- and long-term outcomes. In contrast to NLR and SII, the PNI did not demonstrate a statistically significant impact on survival outcomes in this study. Patients with PNI ≥ 38.8 had a median overall survival of 33 months, while those with PNI < 38.8 had a median survival of 22 months. Although there was a trend toward better survival in the higher PNI group, the difference was not statistically significant (*p* = 0.1). This suggests that while PNI may have some prognostic value, it is not as robust a predictor of survival as NLR or SII in this cohort of patients. The lack of statistical significance may be due to several factors. PNI is calculated based on serum albumin levels and lymphocyte counts, which reflect both nutritional status and immune competence. While these factors are important for overall health and recovery, it is important to note that all indices evaluated in this study—including NLR, SII, and PNI—are based on a limited number of hematological parameters and may not capture the full complexity of the immune and inflammatory responses that play a role in cancer progression and survival. The differential prognostic value observed among these indices may be attributed to the specific components and mathematical formulations of each, which could make certain indices more sensitive to clinically relevant aspects of the systemic inflammatory response [49]. Moreover, PNI was not significantly associated with complication rates (*p* = 0.8) or the severity of complications (*p* = 0.5). These findings are consistent with previous reports suggesting that while PNI may be a useful marker of general health and nutritional status, it may not be a strong predictor of perioperative morbidity in cancer patients. The lack of association with complications also suggests that PNI may not be directly related to the immune and inflammatory processes that influence short-term outcomes following surgery [50].

There are several limitations to this study. This was a retrospective, single-center study, which may introduce selection bias and limit the generalizability of the results. The patient population was relatively small (*n* = 136). Additionally, some patient data were missing, particularly related to complication severity and specific perioperative metrics, which could have influenced the analysis. Another limitation is the heterogeneity of patient factors, such as variations in comorbidities, tumor stage, and the presence of pre-existing inflammatory conditions, which may confound the relationship between immune–inflammatory markers and survival outcomes. While efforts were made to adjust for key clinical variables, residual confounding may still exist. Additionally, the retrospective design inherently limits the ability to establish causality between immune–inflammatory markers and outcomes, restricting conclusions to associations rather than direct effects. Furthermore, the use of NLR, SII, and PNI as prognostic markers, while validated in several cancer types, remains controversial in other clinical settings. These markers are influenced by a range of factors beyond cancer-related inflammation, including infections, autoimmune diseases, and other inflammatory conditions [51]. In our study, we could not fully account for these confounding factors, which may have affected the accuracy of these biomarkers in predicting survival and complications. Additionally, we did not perform a multivariate analysis including all three indices (NLR, SII, and PNI) simultaneously. Our analytic approach focused on evaluating each index individually in relation to survival and postoperative complications. This decision was based on both methodological considerations and the sample size of our cohort. We acknowledge that a multivariate model could provide further insight into the independent prognostic value of each index, and recommend that future studies with larger sample sizes explore this approach. This limitation highlights the need for caution when applying these markers in clinical practice.

To address the limitations of the present study, future prospective research should focus on more robust data to confirm the prognostic utility of immune–inflammatory markers such as NLR, SII, and PNI in patients with adenocarcinoma of the head of the pancreas undergoing pancreaticoduodenectomy. Increasing the sample size and ensuring more comprehensive data collection would improve the power to detect significant associations and minimize the effects of missing data. Future studies should also aim to account for a broader range of confounding factors, including comorbid inflammatory and infectious conditions that could influence the levels of NLR, SII, and PNI. Incorporating biomarkers of inflammation and immune function alongside clinical data could help clarify the specific contributions of immune–inflammatory responses to survival outcomes. Furthermore, longitudinal studies that track changes in these biomarkers over time could provide valuable insights into their dynamic role in cancer progression and treatment response.

In addition to NLR, SII, and PNI, a wide range of other biomarkers—including circulating tumor cells, cell-free DNA, cytokines, and tumor-associated macrophages—have already been extensively studied for their prognostic value in pancreatic cancer [52]. Future research should focus on validating these biomarkers in the specific context of patients undergoing pancreaticoduodenectomy and on integrating them into multi-modal prognostic models alongside established inflammatory indices. These biomarkers could complement existing immune–inflammatory indices by providing a more detailed understanding of the tumor microenvironment and systemic immune responses. Incorporating such markers into future studies could help refine risk stratification models and guide more personalized treatment approaches. Given that our study found limited predictive value of immune–inflammatory markers for postoperative complications, future research should also investigate the integration of these markers with other clinical and surgical factors, such as tumor characteristics, surgical complexity, and perioperative management protocols. Combining immune–inflammatory markers with clinical scoring systems may enhance the ability to predict complications and improve postoperative risk assessment.

## 5. Conclusions

In summary, our findings suggest that immune–inflammatory markers, particularly NLR and SII, have significant prognostic value in patients undergoing pancreaticoduodenectomy for adenocarcinoma of the head of the pancreas. Higher NLR and SII were associated with improved long-term survival, highlighting their potential utility in risk stratification and guiding postoperative management. However, these markers were not significantly associated with complication rates or severity, indicating that their predictive value may be limited to oncological outcomes rather than perioperative morbidity. In contrast, PNI did not emerge as a significant predictor of survival or complications in this study, suggesting that its prognostic value may be more limited in this context. Nonetheless, PNI remains an important marker of nutritional status and may still be useful in the overall assessment of patient health and recovery. Further prospective research with larger sample sizes and more comprehensive data sets is needed to validate these findings and explore the underlying mechanisms linking immune–inflammatory markers to survival and complications in pancreatic cancer patients. Incorporating these markers into clinical practice may help optimize patient outcomes and improve personalized treatment strategies in this high-risk population.

## Figures and Tables

**Table 1:** Patient characteristics for patients categorized by neutrophil-to-lymphocyte ratio (NLR) into two groups: NLR < 2.5 and NLR ≥ 2.5.

Variable	Total (*n* = 136)	NLR < 2.5 (*n* = 49, 36%)	NLR ≥ 2.5 (*n* = 87, 64%)	*p*-Value
Age (years)	66.4 ± 11.7	65.4 ± 12.5	66.9 ± 11.3	0.46
Sex				0.031
F	53 (39%)	25 (51%)	28 (49%)	
M	83 (61%)	24 (28.9%)	59 (71.1%)	
Ethnicity				0.36
Arab	25 (18.4%)	11 (44%)	14 (56%)	
Jewish	111 (81.6%)	38 (34.2%)	73 (65.8%)	
Hypertension				0.87
No	49 (40.5%)	17 (34.7%)	32 (65.3%)	
Yes	72 (59.5%)	26 (36.1%)	46 (63.9%)	
Missing data	15			
Diabetes				0.64
No	82 (67.8%)	28 (34.1%)	54 (65.9%)	
Yes	39 (32.2%)	15 (38.5%)	24 (61.5%)	
Missing data	15			
IHD				0.75
No	103 (85.1%)	36 (35%)	67 (65%)	
Yes	18 (14.9%)	7 (38.9%)	11 (61.1%)	
Missing data	15			
Smoking				0.14
No	90 (84.9%)	34 (37.8%)	56 (62.2%)	
Yes	16 (15.1%)	3 (18.8%)	13 (81.3%)	
Missing data	30			
CA19.9 U/mL				0.92
≤400	104 (85.2%)	36 (34.6%)	68 (65.4%)	
≥400	18 (14.8%)	6 (33.3%)	12 (66.7%)	
Missing data	14			
SII				0.001
<600	54 (39.7%)	45 (83.3%)	9 (16.7%)	
≥600	82 (60.3%)	4 (4.9%)	78 (95.1%)	
PNI				0.68
<38.8	56 (43.1%)	20 (35.7%)	36 (64.3%)	
≥38.8	74 (56.9%)	29 (39.2%)	45 (60.8%)	
Missing data	6			

NLR, neutrophil-to-lymphocyte ratio; IHD, ischemic heart disease; CA19.9, cancer antigen 19.9; SII, systemic immune–inflammation index; PNI, prognostic nutritional index.

**Table 2:** The operative and postoperative outcomes for patients categorized by neutrophil-to-lymphocyte ratio (NLR) into two groups: NLR < 2.5 and NLR ≥ 2.5.

Variable	Total (*n* = 136)	NLR < 2.5 (*n* = 49, 36%)	NLR ≥ 2.5 (*n* = 87, 64%)	*p*-Value
OR time (min)	337 ± 90	335 ± 87	337 ± 92	0.89
Blood loss				0.6
No	19 (14.8%)	5 (26.3%)	14 (73.7%)	
Yes	77 (60.2%)	29 (37.7%)	48 (62.3%)	
Unknown	32 (25%)	10 (31.3)	22 (68.8%)	
ICU				0.155
No	76 (56%)	24 (30.7%)	52 (69.3%)	
Yes	60 (44%)	26 (42.4%)	34 (57.6%)	
ICU stay (days)	7.7 ± 13.3	9.4 ± 10.2	6.5 ± 15.2	0.43
Complication grade				0.22
Clavien I–II	72 (67.3%)	26 (36.1%)	46 (63.9%)	
Clavien ≥ III	35 (32.7%)	17 (48.6%)	18 (51.4%)	
Pancreatic Fistula				0.60
A	37 (54.4%)			
B	29 (42.6%)	12 (32.4%)	25 (67.6%)	
C	2 (2.9%)	10 (34.5%)	19 (65.5%)	
Missing data	68	0 (0%)	2 (100%)	
LOS (days)	18.7 ± 13.6	21.3 ± 15.8	17.4 ± 12.2	0.123
Median OS (months)	29 (14:81)	18 (9:39)	39 (17:100)	0.004

ICU, intensive care unit; LOS, length of stay; OS, overall survival.

**Table 3:** Outcomes for patients categorized by systemic immune–inflammation index (SII) into two groups: SII < 600 and SII ≥ 600.

Variable	Total (*n* = 136)	SII < 600 (*n* = 54, 40%)	SII ≥ 600 (*n* = 82, 60%)	*p*-Value
Median OS (months)	29 (14:81)	20 (9:45)	34 (17:114)	0.001
Complication				0.07
No	29 (21.3%)	7 (24.1%)	22(75.9%)	
Yes	107 (78.7%)	47 (43.9%)	60 (56.1%)	
Complication grade				0.1
Clavien I–II	72 (67%)	27 (38%)	45 (62%)	
Clavien ≥ III	35 (33%)	19 (54%)	16 (46%)	

OS, overall survival; SII, systemic immune–inflammation index.

**Table 4:** Outcomes for patients categorized by prognostic nutritional index (PNI) into two groups: PNI < 38.8 and PNI ≥ 38.8.

Variable	Total (*n* = 136)	PNI < 38.8 (*n* = 61, 43%)	PNI ≥ 38.8 (*n* = 75, 57%)	*p*-Value
Median OS (months)	28 (14:77)	22 (14:60)	33 (14:115)	0.1
Complication				0.8
No	29 (21%)	14 (48%)	15 (52%)	
Yes	107 (79%)	48 (45%)	59 (55%)	
Complication grade				0.5
Clavien I–II	72 (67%)	34 (47%)	38 (53%)	
Clavien ≥ III	35 (33%)	15 (43%)	20 (57%)	

OS, overall survival; PNI, prognostic nutritional index.

## Data Availability

The data presented in this study are available on request from the corresponding author due to legal reasons.
